# Specialist paediatric palliative care program development in the standard-of-care era

**DOI:** 10.1093/pch/pxaf060

**Published:** 2025-07-28

**Authors:** David L Lysecki, Jennifer Callen, Joanna Humphreys, Katherine Sutherland, Cindy van Halderen, Sarah Van Meer, Gregorio Zuniga-Villanueva

**Affiliations:** Department of Pediatrics, McMaster University, Hamilton, Ontario, Canada; McMaster Children’s Hospital, Hamilton Health Sciences, Hamilton, Ontario, Canada; McMaster Children’s Hospital, Hamilton Health Sciences, Hamilton, Ontario, Canada; Department of Pediatrics, McMaster University, Hamilton, Ontario, Canada; McMaster Children’s Hospital, Hamilton Health Sciences, Hamilton, Ontario, Canada; McMaster Children’s Hospital, Hamilton Health Sciences, Hamilton, Ontario, Canada; McMaster Children’s Hospital, Hamilton Health Sciences, Hamilton, Ontario, Canada; McMaster Children’s Hospital, Hamilton Health Sciences, Hamilton, Ontario, Canada; Department of Pediatrics, McMaster University, Hamilton, Ontario, Canada; McMaster Children’s Hospital, Hamilton Health Sciences, Hamilton, Ontario, Canada

**Keywords:** Palliative care, palliative medicine, pediatrics, perinatal care, program development, program evaluation

## Abstract

**Objectives:**

The number of Canadian children living with serious illness is increasing. Access to specialist paediatric palliative care is recognized as essential for these children, their families, and their care providers, and yet programs remain under-resourced or non-existent in much of Canada. Health services planning requires current data. This study examined the initial experience of a specialist program established in 2015 at a tertiary paediatric centre in Canada.

**Methods:**

A prospective database study of referred patients was conducted from 2015 to 2023 (prenatal referrals were first accepted in 2021). Data were collected at referral, consult, and discharge/death/end of pregnancy. Program clinician growth was tracked. The analysis included descriptive statistics and a Kaplan–Meier survival curve.

**Results:**

The program received 650 unique paediatric referrals plus 55 prenatal referrals. The number of patients receiving care annually quadrupled over the course of the study. Two-hundred and twenty-seven children died: 99% with goal-concordant care at end-of-life and most frequently in hospital, although death at home was increasingly common. A rapid increase in program resources was required to meet care needs per modern standards of care.

**Conclusions:**

This study demonstrates that a new paediatric palliative care program was met with demand akin to existing established comparator programs. These results are congruent with the increase in prevalence of children with serious illness and the evolution of care standards to incorporate specialist care provision. These findings can help advocacy and resource planning for modernizing Canada’s paediatric palliative care infrastructure.

## INTRODUCTION

While infant and child mortality in Canada has decreased precipitously over the past century, many Canadian infants, children, and youth are living—often thriving—with a serious illness. “Serious illness” is “a health condition that carries a high risk of mortality and either negatively impacts a person’s daily function or quality of life, or excessively strains their caregivers” (1). The prevalence of serious illness amongst Canadian infants, children, youth, and young adults (age 0 to 25) is 73.1 per 10,000 (2), similar to research from other high-income countries reporting 66.4 to 95.7 per 10,000 children (3–6). Of note, research from England documented an increasing trend from 26.7 per 10,000 in 2001/02 to 66.4 per 10,000 in 2017/18 (3).

**Figure 1.**
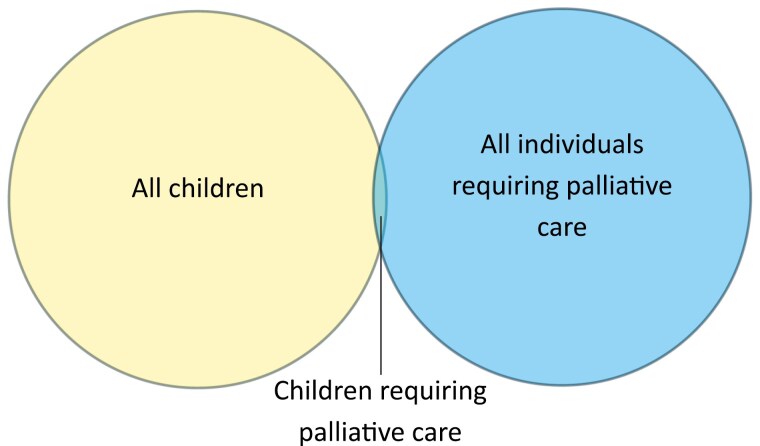
Venn diagram demonstrating the proportion of children requiring palliative care relative to all children and all individuals requiring palliative care.

Children with serious illness benefit from paediatric palliative care (PPC) (7). Their needs and those of their families are specific and complex—physically, psychologically, emotionally, socially, and spiritually. Their care requires expertise in paediatrics, in palliative care, and in the intersection between the two. However, 99% of Canadian children have no palliative care needs (2) and 99% of Canadians with palliative care needs are not children (8) (Figure 1). As a result, few practitioners have experience in the intersection between the two (9). Dedicated PPC resources therefore represent a cornerstone of high-quality care, and play critical roles in direct and indirect care, education, training, resource generation, research, advocacy, policy creation, and leadership. Inclusion of specialist PPC teams in patient care, including the newer field of perinatal palliative care (PnPC), has been associated with improved patient outcomes and system cost-effectiveness (7,10–15). Consequently, access to interdisciplinary specialist PPC teams is increasingly being recommended in national and international guidelines (16–21), in disease-specific care pathways (22), and in referral trigger criteria (23–26).

Access to PPC resources, however, remains inequitable for Canadian patients, families, and their care providers. To meet this challenge, advocacy, resourcing, and care modelling are required. In the context of rising prevalence of patients and evolving standards-of-care, current data is required to inform health services planning. This study sought to examine the initial experience of a Canadian tertiary specialist PPC program founded in 2015. Focused analyses include end-of-life outcomes, the new field of PnPC, and program growth.

## METHODS

This was a prospective database study approved by the Hamilton Integrated Research Ethics Board (#2203). The requirement of consent to participate was waived as risks to anonymous data collection were low and referrals often came at highly sensitive periods for families.

### Setting and intervention

The Quality of Life & Advanced Care (QoLA Care) program was initiated in November 2015 as the specialist PPC program at McMaster Children’s Hospital, a 165-bed tertiary paediatrics hospital in Hamilton, Ontario, Canada with a catchment of 2.3 million persons (~500,000 age less than 19 years). QoLA Care accepted referrals for paediatric patients with serious illness. There were no automatic triggers or mandatory referral criteria. Referrals for patients over 18 years of age were assessed case-by-case. The program began accepting prenatal referrals in February 2021. There were no local or affiliated paediatric hospice facilities.

Once patients were accepted, the program provided 24/7 availability, consultative care to inpatients, clinic visits and virtual care to outpatients, and shared care models in home and community settings as available. When no community resources were available to oversee end-of-life care in the home/community, the program functioned as the most responsible team. Patients active in the program were seen as clinically indicated, ranging from multiple times per day to annual assessments of quality of life and goals of care, and all had 24/7 access. Patients leaving the region were transitioned to other PPC programs. Patients reaching age 18 remained in the program if death was anticipated within one year, otherwise they were transitioned out of paediatric services. Patients were discharged from the program if there was agreement between the family and clinicians that they no longer met referral criteria (i.e., at low risk of mortality in childhood, no active quality of life concerns). The program offered grief support and bereavement transitional care to families of children within the program at the time of death, but did not have a formal grief and bereavement program, and did not receive referrals for bereavement care only.

### Data collection

The inclusion criterion was referral to the program; there were no exclusion criteria. The paediatric dataset included data of paediatric patients collected at referral, consult, and discharge/death (Appendix A) from November 2015 through November 2023. Each patient had a unique entry; therefore, if patients were re-referred after discharge, their data were added to their initial entry. A maternal/fetal dataset was added in February 2021, which included data collected at referral, consult, and the end of the pregnancy (Appendix A). If the pregnancy resulted in a liveborn infant, the infant was then also added to the paediatric dataset.

### Analysis

Results are descriptive. For patients active at study end, days of palliative care were measured from the consult to the cut-off date. We used Kaplan-Meier methodology to generate a survival curve.

## RESULTS

### Paediatric dataset

**Figure 2.**
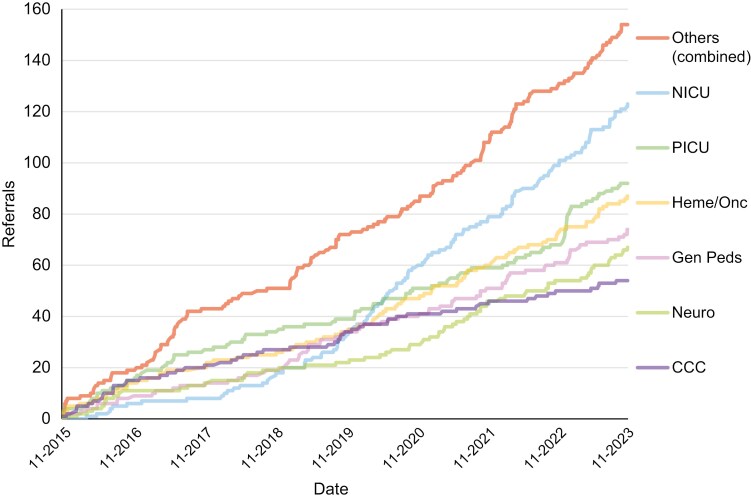
Referring service over time, pediatric dataset.

QoLA Care received referrals for 650 unique infants, children, youth, or young adults from 25 unique specialties (Table 1 and Figure 2). 623 patients had consultations (Table 2). The remaining were pending (*n* = 8), redirected to a more appropriate service (*n* = 8), retracted by referring team (*n* = 6), refused by family (*n* = 3), or died before consultation (*n* = 2). Thirty-nine patients (6%) had a sibling who also had a serious illness (alive or deceased).

**Table 1.** Referral source, paediatric and prenatal datasets

	Paediatric *n* = 650	Maternal/Fetal *n* = 55
**Referring service**	**(*n*, %)**	**(*n*, %)**
Neonatology	124 (19)	11 (22)
Critical care	91 (14)	
Hematology/oncology	88 (14)	
General paediatrics	74 (11)	
Neurology	67 (10)	<5
Chronic complex care	55 (8)	
Orthopedic surgery	20 (3)	
Maternal-fetal medicine	20 (3)	36 (71)
Neuromuscular	19 (3)	
Physiatry	18 (3)	
Gastroenterology	17 (3)	
General surgery	16 (2)	
Genetics/metabolics	15 (2)	<5
Cardiology	5 (1)	
Other^*^	23	<5

^*^Other services with < 5 referrals included: Immunology, Paediatric Palliative Care, Neurosurgery, Family Medicine, Nephrology, Developmental Paediatrics, Infectious Diseases, Psychiatry, Adolescent Medicine, Rheumatology, Respirology, and Midwifery.

**Table 2.** Patient demographics at consultation and death, paediatric dataset

	Consults (*n* = 623)	Deaths (*n* = 227)
**Age at consult/death**	**(*n*, %)**	**(*n*, %)**
Prenatal	28 (4)	N/A
<1y	182 (29)	101 (44)
1 to 4 y	94 (15)	36 (16)
5 to 9y	107 (17)	35 (15)
10 to 14y	107 (17)	25 (11)
15 to 18y	95 (15)	24 (11)
>19y	10 (2)	6 (3)
**Sex**	**(*n*, %)**	**(*n*, %)**
Female	305 (49)	109 (48)
Male	318 (51)	118 (52)
**Primary diagnostic category**	**(*n*, %)**	**(*n*, %)**
CNS condition	168 (27)	48 (21)
Chromosomal/multi-organ syndrome	148 (23)	53 (23)
Cancer	87 (14)	62 (27)
Metabolic/biochemical	37 (6)	13 (6)
Neuromuscular	35 (6)	11 (5)
Perinatal	34 (5)	20 (9)
Infection	33 (5)	<5
External	19 (3)	<5
Musculoskeletal	13 (2)	<5
Cardiovascular	15 (2)	8 (4)
Immunologic	8 (1)	<5
Renal	7 (1)	<5
Gastrointestinal	7 (1)	0 (0)
Respiratory	5 (1)	<5
Hematologic	3 - 6 (1)	<5
Other	<5	0 (0)
**Location of consult/death**	**(*n*, %)**	**(*n*, %)**
Inpatient unit (non-ICU)	194 (31)	43 (19)
Clinic	164 (26)	<5
NICU (including care-by-parent)	102 (16)	46 (20)
PICU	77 (12)	40 (18)
Virtual	59 (9)	N/A
Home	22 - 26 (4)	59 (26)
Labour and delivery	<5	13 (6)
Residential hospice	0 (0)	12 (5)
Emergency department	0 (0)	9 (4)
Adult ICU	0 (0)	<5

One-hundred and ninety-one patients (30%) were alive and actively being cared for in the program at study end. These patients had been in the program for a median of 739 days (range: 2 days to 7.5 years) as of the cut-off date. Two-hundred and five patients (33%) had been discharged alive after a median of 139 days (range: 2 days to 6.5 years). Nineteen patients (3%) had been transitioned to adult care and 13 (2%) to another region’s PPC program.

**Figure 3.**
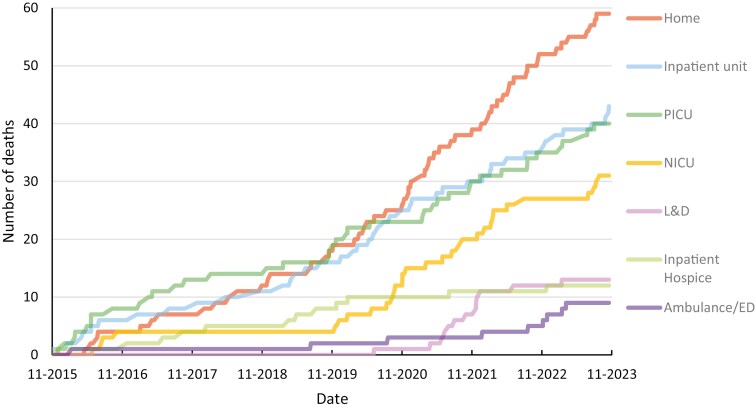
Location of death over time, pediatric dataset.

Two-hundred and twenty-seven patients (36%) died after a median of 51 days in the program (range: 1 day to 7.5 years) (Table 2). Eighty-eight patients (39%) died within 30 days of consultation, 96 (42%) between 31 days to 1 year, and 43 (19%) after 1 year. Seventy-one patients (31%) had withdrawal of one or more cardiac, pulmonary, or renal life-sustaining therapies, 62 of whom died in the hospital (87%). Two-hundred and twenty-four patients (99%) had care at the time of death consistent with their most recently documented goals. Seventeen patients (7%) underwent attempts at cardiopulmonary resuscitation at the time of death. Figure 3 illustrates the trend in location of death over time. Seventy-one patients (31%) died in community settings (home or residential hospice). The most responsible physician for this care came from QoLA Care (*n* = 32; 46%), community palliative care (*n* = 24; 34%), general paediatrics (*n* = 7; 10%,) and family medicine (*n* = 7; 10%).

**Figure 4.**
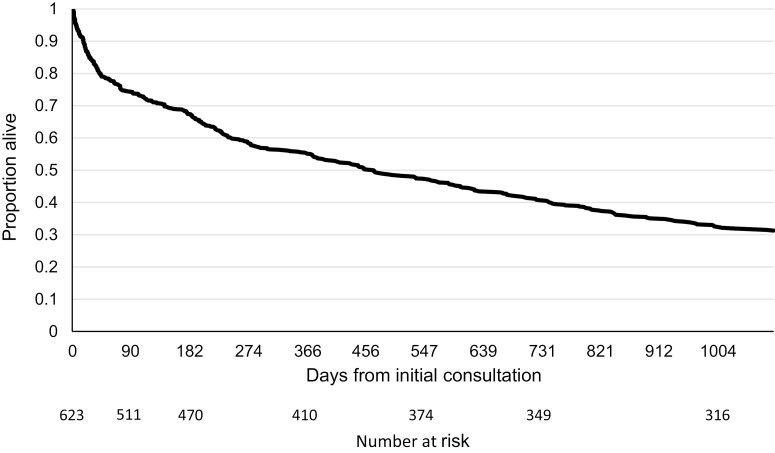
Kaplan-Meier survival curve by days from initial consultation.

All-in-all, the program provided 280,390 patient-days of 24/7 access to specialist PPC over an 8 year period. Figure 4 demonstrates a Kaplan–Meier survival curve for patients as a function of days from initial consultation.

### Maternal/fetal dataset

The program received 55 prenatal referrals over 34 months (Table 1) and completed 49 consults. The remaining were retracted by the clinical team (*n* = 3), declined by family (*n* = 2) and pending (*n* = 1). Location of consult included virtual (*n* = 26; 53%), outpatient clinic (*n* = 14; 29%), inpatient unit (*n* = 8; 18%), and home (*n* < 5). Fetal diagnostic categories included chromosomal/multi-organ syndromes (*n* = 17; 35%), central nervous system disorders (*n* = 11; 22%), renal anomalies (*n* = 6; 12%), cardiovascular anomalies (*n* = 6; 12%), as well as metabolic, musculoskeletal, or hematologic conditions, and complications of pregnancy (*n* < 5 each). Pregnancies resulted in live births (*n* = 32; 66%), stillbirths (*n* = 12; 27%), and termination or transition to another center (*n* < 5 each).

### Program growth

**Figure 5.**
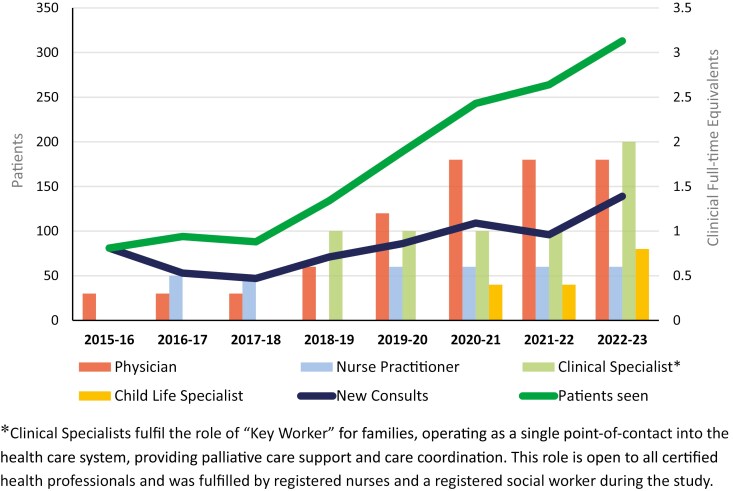
Program clinical full-time equivalents versus annual patient volumes and new referrals.

The annual number of new consults fluctuated but increased over the study, while the annual number of patients in the program increased steadily (Figure 5). The program began with temporary funding for a 0.5 full-time equivalent (FTE) physician and grew to entail 6.4 FTEs of an interdisciplinary team. A portion of physician FTE was dedicated to non-clinical deliverables (e.g., teaching, research, administration), resulting in a total team clinical FTE complement of 5.2. Permanent funding was established for physicians in July 2018 and for other professionals in October 2023. The ratio of patients seen per year to clinician FTE at the onset of that year was 270 in year 1, followed by 117.5, 110, 83.8, 67.5, 63.9, 69.5, and finally, 60.2.

## DISCUSSION

The results of this study can be contextualized through comparisons of past initial experiences for other Canadian programs. CHEO is a similarly-sized institution which initiated a program in 1999 and received 341 referrals over its initial 9 years (27), as compared to 705 referrals over 8 years in this study. Canuck Place Children’s Hospice opened in 1995, associated with British Columbia Children’s Hospital, and reported providing direct care to 649 children over its first 15 years (28), as compared with 623 children over 8 years in this study. This pattern of increased initial demand has also been reported in the United States: a study program received nearly four times the number of referrals in their first year (2011/12) than had been anticipated based on reports from the published literature (29).

In addition to historical data from new programs, cross-sectional data from established programs can be another source of comparison. The most recent assessment of PPC programs across Canada occurred in 2012 (30). Of 13 programs, the median program age was 13 years (interquartile range, IQR: 8 to 17), median clinician FTE was 2.65 (IQR: 1.3 to 4.8), median annual patient volumes were 67 (IQR: 54 to 110), and programs had a median patients-per-clinician FTE ratio of 35.4 (IQR: 20 to 52). In the final year of this study, the program was 8 years old, had a clinical FTE of 5.2, saw 313 patients, and had a patients-per-clinician FTE ratio of 60.2. This program was larger and saw more patients at a higher patients-per-clinician FTE ratio in its eighth year than many mature programs in 2012. Contemporaneous comparisons with these programs, however, cannot be made without more recent data; many of these programs have also likely experienced growth in patient volumes and staff complement over the past decade. With respect to end-of-life outcomes, death occurred at home and in hospice at similar rates to those reported by established Canadian PPC programs without an affiliated hospice facility (26% vs. 23.1% and 5.3% vs. 4.6%, respectively) (30).

Program workload is dictated not only by the number of patients but also by the duration of their care. The survival curve was consistent with published PPC survival curves from North America (28,31), demonstrating a >50% survival of eligible patients at 1 year. Survival for many PPC conditions, both curable and incurable, has improved over recent decades. Twenty-eight percent of children in this study (173/623) were eventually deemed at low risk for death in childhood and discharged from the program. Prognostication in paediatric serious illnesses remains notoriously difficult (32), and referral in cases of uncertainty is appropriate.

Overall, the results of this study appear to indicate rapid uptake and utilization of a new PPC program consistent with modern standards of PPC practice. The American Academy of Pediatrics (AAP) guidelines for PPC and Hospice Care include the statement: “Although programs often start with only a few team members, mature teams should include physicians, nurses, social workers, case managers, spiritual care providers, bereavement specialists, and child life specialists” (16). The findings of this study challenge the notion of programs “starting small” in the modern context of PPC. This program was met with demand akin to established programs and required rapid growth. Core offerings (such as 24/7 on-call support) were necessary to achieve the findings observed in this study, particularly the provision of end-of-life care in home settings (whether as the most responsible team or to provide specialist support to community providers). To account for this volume of work, staffing increased by 12.8 times over the course of the study. At study end, the patients-per-clinician FTE ratio remained above the upper quartile of Canadian programs in 2012 (30), and this program continued to lack social work, spiritual care, and bereavement specialist resources as compared with the AAP recommendation.

There were patient groups, however, that appeared to represent exceptions to rapid early utilization, including fetuses, infants in the neonatal intensive care unit, and patients with solid-organ failure. In the early years, there were no prenatal referrals and referrals from neonatology were few. Over the course of this study, these referrals became a pivotal driver of program growth for both end-of-life and chronic palliative care, befitting the fact that many paediatric serious illnesses are present from the time of conception or birth and many paediatric deaths occur before age 1 year (8). This shift coincided with the addition of a nurse practitioner to the PPC team who had previously spent 20 years working in neonatology, highlighting the importance of relationships and trust in PPC program evolution particularly in areas where care pathways and standards-of-care are not as clearly defined (the term “perinatal hospice” did not appear in the literature until 1997 (33) and “perinatal palliative care” in 2006 (34).). Patients with single-organ failure (primary cardiac, respiratory, hepatic, or renal conditions) also composed a small fraction of referrals. Many of these patients were referred from the quaternary centre, having been transferred there for solid organ transplantation and being referred back for acute community-based end-of-life care. We postulate that referrals for these groups may be concentrated in centres which offer organ transplantation; however, the return of these patients needing acute community-based end-of-life care signals the potential for early referral pathways and the importance of collaboration between centres.

The most important limitation of this study is the absence of patient- and family-reported measures to examine the experience of care. This research is underway using the Quality of Children’s End-of-Life Care Instrument (35). Similarly, research is being conducted on the regional impact on children’s healthcare utilization and cost near end-of-life (15). Recent data from comparator programs across Canada is a critical need to understand and plan for the equitable delivery of PPC nationally.

The prevalence of Canadian children with palliative care needs is rising. Patients, their families, and care providers deserve equitable access to specialist PPC resources, including direct and indirect care, education, training, resource generation, research, and advocacy. The findings of this study suggest that new and expanded resources should expect large, unmet demand; put simply, *if you build it, they will come*. This study indicates that established PPC programs (adjusted for catchment population and program scope) provide more realistic predictors for new programs than do historical experiences of new programs. This study refutes the notion of “starting small” in the modern context. To meet modern care standards, an interdisciplinary approach is required and should be considered in resource planning. A targeted approach may be required for the relatively new field of PnPC, which represents a high volume of acute palliative care and can represent early identification for many with chronic needs. Standardized assessment of the quality of palliative care received was not assessed in this study and would support a better understanding of the relationship between program resources and patient/family experience and support continuous quality improvement.

## APPENDIX A: Research datasets

**Table:**

Paediatric dataset		
Referral	Consult	Discharge
Date of referral	Date of consult	Date of discharge
Referral source	Location of consult	Discharge status (alive/deceased)
If no consult,	Age at consult	Days of palliative care
-Reason	Sex	If deceased,
Date of re-referral	Primary disease category	-Age at death
	Sibling in program or deceased?	-Location of death
	Date of re-consult	-Withdrawal of cardiac/pulmonary/renal support?
		-Cardiopulmonary resuscitation?
		-CPR/No CPR in keeping with latest goals of care?
		Date of re-discharge
**Maternal/fetal dataset**		
**Referral**	**Consult**	**Discharge**
Date of Referral	Date of Consult	Outcome of pregnancy
Referral Source	Location of consult	
	Primary disease category	
